# Direct Isolation, Culture and Transplant of Mouse Skeletal Muscle Derived Endothelial Cells with Angiogenic Potential

**DOI:** 10.1371/journal.pone.0001753

**Published:** 2008-03-12

**Authors:** Nicholas Ieronimakis, Gayathri Balasundaram, Morayma Reyes

**Affiliations:** Department of Pathology, University of Washington, Seattle, Washington, United States of America; Monash University, Australia

## Abstract

**Background:**

Although diseases associated with microvascular endothelial dysfunction are among the most prevalent illnesses to date, currently no method exists to isolate pure endothelial cells (EC) from skeletal muscle for *in vivo* or *in vitro* study.

**Methodology:**

By utilizing multicolor fluorescent-activated cell sorting (FACS), we have isolated a distinct population of Sca-1^+^, CD31^+^, CD34^dim^ and CD45^− ^cells from skeletal muscles of C57BL6 mice. Characterization of this population revealed these cells are functional EC that can be expanded several times in culture without losing their phenotype or capabilities to uptake acetylated low-density lipoprotein (ac-LDL), produce nitric oxide (NO) and form vascular tubes. When transplanted subcutaneously or intramuscularly into the tibialis anterior muscle, EC formed microvessels and integrated with existing vasculature.

**Conclusion:**

This method, which is highly reproducible, can be used to study the biology and role of EC in diseases such as peripheral vascular disease. In addition this method allows us to isolate large quantities of skeletal muscle derived EC with potential for therapeutic angiogenic applications.

## Introduction

The skeletal muscle is the organ with major microvascular mass [1]. Consequently there are many diseases associated with dysfunctional EC and/or microvascular tissue of the skeletal muscle including atherosclerosis, hypertension, diabetes, obesity, and peripheral vascular disease (PVD) [2]–[8]. PVD has become in recent years one of the most prevalent illnesses among Americans affecting an estimated 29% of the population older than 70 years [9]. In addition muscle diseases including muscular dystrophies and inflammatory diseases are also associated with microvascular dysfunction of the skeletal muscle [10]–[13] . Yet the study of endothelial function in all these diseases associated with microvascular dysfunction in the skeletal muscle has been restricted by the ability to efficiently characterize and isolate EC *in vivo* and expand them *in vitro*. Nonetheless the phenotype of endothelial cells from skeletal muscles has not been well characterized. Furthermore to our knowledge, a procedure that directly isolates pure populations of endothelial cells (EC) from skeletal muscle does not presently exist.

In the literature protocols utilizing culture methods, sorting based on acetylated low-density lipoprotein (ac-LDL) uptake or expression of CD31 have been described for isolating EC from non-muscle tissues [14]–[17]. However, such methods inherently isolate a heterogeneous population of cells some of which are endothelial. This is due to the non-specificity of selective culturing or sorting when utilizing only one marker such as CD31 which is not solely expressed by EC [18]. Though ac-LDL uptake has proven valuable in functional assays it is not exclusive to EC but also a property of macrophages [19]. Despite application in various tissues, none of these methods have been applied to study endothelial cells from skeletal muscles.

Here we first characterize the phenotype of EC from skeletal muscles by staining mouse muscle tissue for Sca-1, CD31 and CD34. We then used these markers to develop a method utilizing multicolor flow cytometry to exclusively sort muscle derived venular, arterial and capillary EC which are all positive for Sca-1, CD31 and CD34 while excluding hematopoietic cells which express the common leukocyte antigen CD45. We can FACS-sort these endothelial cells from mouse skeletal muscles and culture them for more than 80 days. In culture these muscle EC retain endothelial functions and upon transplantation demonstrate robust angiogenic capacity. Thus this technique can be used to study pure populations of muscle EC and to develop angiogenic therapeutic strategies.

## Materials and Methods

### Immunofluorescent and immunohistochemical Tissue Staining

To identify the skeletal muscle vascular phenotype, pectoralis, triceps, tibialis anterior, gastronemous and quadriceps tissue sections from 6–12 month old (MO) C57BL6 mice fixed with 2% Paraformaldehyde (PFA) were stained for smooth muscle actin (SMA), Sca-1 and CD34, then costained for CD31. Slides were also stained with 4′,6-Diamidino-2-phenylindole dihydrochloride (DAPI), purchased from Vector labs (Burlingame, CA). Cy3 conjugated monoclonal anti-SMA antibody (Sigma, St. Louis, MO) was used at 1∶400. Purified polyclonal rat anti-CD34 and biotinylated polyclonal anti-Sca-1 were purchased from BD Pharmingen (San Jose CA) and diluted at 1∶50 and 1∶200. Anti-rat Alexa 594 (Invitrogen, Carlbad, CA) and Texas Red conjugated Avidin (BD Pharmingen, San Jose, CA), were diluted at 1∶1000 and 1∶500. FITC conjugated anti-CD31 (BD Pharmingen, San Jose CA) was used at 1∶50. Prior to staining with the biotinylated anti-Sca-1, tissue was treated with avidin/biotin block (Vector Burlingame, CA). All antibodies were diluted in phosphate buffered saline (PBS)+1% bovine serum albumin (BSA). Controls with only secondary antibodies were included for all staining described in this manuscript.

### Staining of Freshly FACS-sorted cells on cytospins

Freshly FACS-sorted cells fixed with 2% PFA were cytocentrifuged onto microscope slides for 5 minutes at 400 g rcf. Immunoperoxidase staining was done using a polyclonal rabbit anti-vWF antibody (Dako, Carpinteria, CA) at a 1∶200 dilution followed by a biotinylated anti-rabbit antibody at 1∶200 and treatment with the Vectastain ABC kit and 3,3′-diaminobenzidine (DAB) substrate kit according to manufacturers protocol (Vector Burlingame, CA). Avidin/biotin block was administered prior to staining with the biotinylated anti-rabbit antibody.

### Staining of cultured muscle EC

Immunofluoroscence staining of cultured muscle EC was done using a polyclonal rabbit anti-vWF antibody (Dako, Carpinteria, CA) at a 1∶200 dilution followed by anti-rabbit Alexa 594 conjugated antibody (Invitrogen, Carlbad, CA) at 1∶1000. Polyclonal rabbit anti-eNOS (Abcam, Cambridge MA) was used at 1∶100 followed by anti-rabbit Alexa 647 antibody (Invitrogen, Carlbad, CA) at a 1∶800 dilution. Cells were also stained with DAPI to visualize the nuclei.

### Staining of transplanted tissues

Tissue sections were fixed with 2% PFA and stained separately for vWF, CD31 and SMA then co-stained with DAPI. Anti-vWF was used as previously described but followed with anti-rabbit Alexa 647 antibody (Invitrogen, Carlbad, CA) diluted at 1∶800. Purified rat monoclonal anti-CD31 (eBioscience, San Diego, CA) was diluted at 1∶50 followed by anti-rat Alexa 647 antibody (Invitrogen, Carlbad, CA) diluted at 1∶800. Monoclonal FITC conjugated anti-SMA (Sigma, St. Louis, MO) was diluted at 1∶500. All immunofluorescence described in this manuscript was detected using a Zeiss Axiovert 200 fluorescent microscope (Thornwood, NY). Photographs were taken with an onboard monochrome AxioCam MRm camera and colored using Adobe Photoshop (San Jose, CA).

### Cell Isolation

All mice used in this study were C57BL6, predominantly male, ages ranging from 1-25 months old (n = 50). Gender comparisons were initially conducted with 2 month old (MO) mice. As there was gender variation in the percentage of muscle EC found in 2 MO mice, subsequent isolations for muscle groups were conducted only in males. Graphs, statistical analysis, and error bars were created using Microsoft Excel (Redmond, WA) by standard deviation and student t-test.

For each FACS analysis skeletal muscles including triceps, pectorals and all limb muscles were harvested aseptically from multiple mice. With the exception of the analysis conducted for each muscle group, skeletal muscles from each mouse were pooled and treated as individual samples. Muscle was minced with dissecting scissors into small pieces ≤3 mm then digested with a final concentration of 1.2units/ml Dispase II, 2 mg/ml Collagenase type IV (Worthington, Lakewood, NJ) and 2 mM CaCl_2_ in PBS for 45 minutes at 37°C to release mononuclear cells. Every 15 minutes samples were pipetted first with a 25 ml and then a 5 ml serological pipet to fracture clumps and dissociate mononuclear cells. The digestion was neutralized by adding twice the original volume HAM'S/F10 supplemented with 15% horse serum (all from HyClone, Logan, UT). To eliminate connective tissue and fibers, samples were passed through 70 µm followed by 40 µm nylon cell strainers with a filter area of 3.14 mm^2^ (BD Falcon, Franklin Lakes, NJ). Without subsequent washing each sample containing 40 ml was filtered into 50 ml standard polypropylene conicals then centrifuged at 300 g rcf for 5 minutes and resuspended in hemolytic buffer (155 mM NH_4_Cl, 10 mM KHCO_2._ and 0.1 mM EDTA in H_2_O) for 5 minutes at room temperature. Cells were counted, centrifuged once more and resuspended in 1 ml PBS (HyClone, Logan, UT) then transferred to 1.7 ml Eppendorf tubes for staining.

### Flow Cytometry and Cell Sorting

For FACS muscle mononuclear cells were stained with fluorescently conjugated monoclonal antibodies for Sca-1, CD31, CD34 and CD45. Antibody/fluorophore combinations used were anti-Sca-1 APC, anti-CD34 PE Cy7 (eBioscience, San Diego, CA), ant-CD31 FITC or anti-CD31 PE, and anti-CD45 PerCP (BD Pharmingen, San Jose CA). The following PE and FITC conjugated monoclonal antibodies were used separately on different staining tubes from the same mouse muscle preparations: anti-Syndecan 4 PE (BD Pharmingen, San Jose CA), anti-AC133 FITC, anti-NCAM PE and anti-CXCR4 PE (eBioscience, San Diego, CA). Antibodies were diluted and combined in PBS+0.3%BSA at a ratio of 600 ng of each antibody per 10^6^ cells (3 µl from 200 µg/ml stock), except for Sca-1 APC which was added at 300 ng per 10^6^ cells due to its strong avidity. Single color controls stained with 200 ng of each antibody/fluorophore were arranged with 10^5^ cells per control along with one unstained control containing only cells. Samples and controls with antibodies in PBS+0.3% BSA were incubated on ice in the dark for 45 minutes. Cells were then washed, centrifuged at 300 g rcf for 5 minutes and resuspended in PBS+0.3% BSA.

FACS-sorting was performed using a BD Aria equipped with fiber optic guided solid state 488 nm, 633 nm and 407 nm diode lasers, 11 bandpass filters and Diva software for compensation and initial analysis. Utilizing the 488 nm blue laser the bandpass filter range for FITC detection was 515–545 nm, 575–601 nm for PE, 675–695 nm for PerCP and 780–840 for PE Cy7. The 633 nm red laser was used for APC detection within a 660–680 nm filter. Potential spectral overlap between color filters and fluorophores was corrected by software compensation of single color controls. Initially forward side scatter-area vs. height (FSC-A vs. FSC-H) and side scatter-area vs. height (SSC-A vs. SSC-H) parameters were used to gate out duplets or any large granulated events that could represent cellular debris or aggregation. Next hematopoietic cells were excluded by gating out CD45^+^ cells. Subsequently CD45^−^, Sca-1^+^ and CD31^+^ cells were selected as muscle EC for sorting. Positive events and gates were determined by comparing fluorophore signal intensities between the unstained control and each single antibody/fluorophore control. FACS-sorted cells were collected in medium for culture, RLT buffer (Qiagen, Valencia, CA) for reverse transcription or quantitative PCR (RT-PCR, Q-RT-PCR) or 2% PFA for direct immunohistochemistry staining via cytocentrifugation onto glass slides. Post sorting FACS data analysis and graph generation was done on FlowJo v7.13 (TreeStar, Ashland, OR).

### Culture and Viability

Sorted muscle EC were seeded at a density of 850 cells/cm^2 ^on uncoated standard polystyrene tissue culture dishes (BD Falcon, Franklin Lakes, NJ) using high glucose Dulbecco's Modified Eagle's Medium (DMEM) supplemented with 10% fetal calf serum (FCS), 100units/ml Penicillin with 100 µg/ml Streptomycin (HyClone, Logan, UT) and 10 ng/ml VEGF (R&D, Minneapolis, MN). Cultures were incubated at 37°C, 5%CO_2_ and 5%O_2_. Media was initially changed at day three and subsequently replaced every four days or with every passage to a new dish.

To estimate cell death within the first 24 hours of culture, fresh muscle mononuclear cells were stained with anti-CD45 PerCP, anti-Sca-1 APC, and anti-CD31 FITC as previously described but with DAPI (Sigma, St. Louis, MO) at a final concentration of 10 µg/ml in PBS+0.3% BSA. Dying or injured cells resulting from cell preparation were excluded from sorting as DAPI^+^ cells detected by the BD Aria violet 407 nm laser and 450–500 nm filter. 1000 viable endothelial cells defined as Sca-1^+^, CD31^+^, CD45^−^ and DAPI negative were directly deposited into each well of a 24 well plate by flow cytometry automated cell deposition. The average cell death was quantified 24 hours after culture by staining each well and supernatant with DAPI (n = 4). DAPI positive cells were counted in the supernatant as well as in the adherent fraction on the plate.

### In vitro functional assays

EC functional capabilities for vascular tube formation, ac-LDL uptake and nitric oxide (NO) production were assayed at various times up to 80 days in culture. For vascular tube formation cells were seeded at a density of 26,000 cells/cm^2 ^on tissue culture dishes coated with pure growth factor reduced matrigel (BD, Franklin Lakes, NJ) and incubated overnight at 37°C, 5%CO_2_ and 5%O_2_. Controls without matrigel were seeded in parallel.

Acetylated low-density lipoprotein (ac-LDL) uptake was performed by adding 10 µg/ml ac-LDL labeled with rhodamine (Biomedical Technologies Inc, Stoughton, MA). Nitric Oxide (NO) activity was measured using the nitric oxide synthase detection kit by Sigma (St. Louis, MO) following manufacturer's protocol. NO production was blocked with 6 µM of Nω-Nitro-L-arginine methyl ester hydrochloride (L-NAME), (Sigma, St. Louis, MO). Samples for both assays were incubated at 37°C for a minimum of 3 hours.

### In vivo syngeneic transplant studies

Three different types of transplant studies were conducted; cultured cells injected with matrigel, cultured cells directly injected intramuscularly and freshly FACS-sorted cells directly injected intramuscularly. Initially the *in vivo* angiogenic potential of cultured EC was examined via the standard dorsal matrigel plug assay modified to include matrigel plug insertion over the tibialis anterior (TA) in parallel with direct injection of plain muscle EC into the TA [20]. Muscle EC derived from 25 MO male C57BL6 mice were harvested at 70 days in culture for transplant with and without reduced growth factor matrigel (BD, San Diego, CA). 1×10^6^ cells resuspended in 200 µl of 50% matrigel with PBS were injected subcutaneously over the dorsum. 10^6^ cells resuspended in 35 µl 75% matrigel with PBS were injected subcutaneously over the TA. Special attention was placed to prevent insertion of the needle in the TA muscle. 10^6^ cultured EC were resuspended in 35 µl PBS and directly injected into the TA of individual mice. In this case approximately 1 mm of the needle was inserted deep in the muscle.

Freshly sorted EC defined as Sca-1^+^, CD31^+^, CD45^−^ along with Sca-1^−^, CD31^− ^, CD45^−^ control cells were harvested from 2 MO C57BL6 mice and also transplanted via direct injection into the TA. In this experiment DAPI was again administered to cell preparations prior to sorting at a final concentration of 10 ng/ml in PBS+0.3% BSA to exclude dying cells. Thus for the transplant of freshly sorted cells only DAPI negative, e.g. viable cells, were selected for injection. The direct transplant of freshly sorted cells was conduced under the same conditions as cultured cells with 10^6^ EC and control cells being resuspended in 35 µl PBS then directly injected into the TA of individual mice.

For all transplants donor cells were stained with the fluorescent membrane dye PKH26 (Sigma, St. Louis, MO) according to manufacturers instructions. PKH26 stained cells were washed and resuspended for transplant into 2 MO male C57BL6 mice (n = 8). All mice were sacked 14 days post injection and analyzed. Once the dermal tissue was removed matrigel plugs were visible while remaining attached to the TA and dorsal skin. TAs and the dorsal plug were removed and sectioned. Euthanasia and animal injections were in compliance with local guidelines and protocols approved by the Institutional Animal Care and Use Committee.

### Reverse Transcription PCR

RNA was extracted from cells using the Qiagen (Valencia, CA) Rneasy kit following manufacturer's protocol. First strand cDNA was synthesized using the High Capacity cDNA synthesis kit from Applied Biosystems (Foster City, CA) per manufacturer's instructions. PCR for specific target genes was performed using the following thermal cycling conditions, 95°C -7′ initial activation followed by 94°C-30”;57°C-30”;72°C-45”, for 35 cycles using the Immomix PCR mastermix from Bioline (Randolph, MA). Primer sequences for each respective target gene are listed in table 1.

**Table 1:** Primers

***RT-PCR Primer Sequences***		
Target Gene	Forward Primer	Reverse Primer
AC133	CCA TGC AGG AGG AAG TGC TT	TGC TCC ACT ACA TAG TCA ATT TGC T
c-met	TTA TTC ATG GGC CGG CTT AA	TGG TGA TCT TCT TTC CTG TGA CA
CD36	CAT CCA AAT GAA GAT GAG CAT AGG	CCA TGC CAA GGA GCT TGA TT
CXCR-4	GCC ATG GAA CCG ATC AGT GT	ACA GGT GCA GCC GGT ACT TG
Msx-1	GGC CTC TCG GCC ATT TCT	TAC TGC TTC TGG CGG AAC TTG
Pax3	CGC TGT CTG TGA TCG GAA CA	TCT GCT CCT GCG CTG CTT
Pax 7	GCT GCT GAA GGA CGG TCA CT	TCG ATG CTG TGT TTG GCT TTC
NCAM	TGC TCG TGT GTC CTC CTT GA	GCT TGG CAG CAA CTG ACC AT
MyoD	GCT ACC CAA GGT GGA GAT CCT	GGC GGT GTC GTA GCC ATT
Myf-5	CAA GCT TTC GAG ACG CTC AAG	TGT CAA AGC TGC TGT TCT TTC
Syndican 3	AAC AAA GGC CGC CAT GAA	CCC CGA GTA GAG GTC GTC TAG TT
Syndican 4	CCC GGA GAG TCG ATT CGA	TCC AGT TCC TTG GGC TCT GA
Flk-1	AAA GAC AAC GAG ACC CTG GTA GAA	CAA TGA CAA GAA GGA GCC AGA A
Tie 1	TGT TCG TGG CCT CAA TGC TA	TCG GAT ACA CAC CAA GGC TAA A
Tek	GGA ACC TGA CCT CGG TGC TA	CCC TGA ACC TTA TAC CGG ATG A
vWF	GAT GTC CAG CTC CCC TTC CT	AGG CGT TTC CGA AGT CTA CCA
Flt-1	GGT GAA GAT TTG CGA CTT TGG	CCG CAT GCC TTC CTT CAG
CD34	CTT CTG CTC CGA GTG CCA TT	GCC AAG ACC ATC AGC AAA CAC
Ephrin B2	TGG AGG GCC TGG ATA ACC A	ACT TCG GAA CCC AGG AGA TTG
Eph B4	GAG GGC TGC GTC CTG ATG T	TCT GCG CCC TTC TCA TGA TA
P-Selectin	CCC TCC CCA TGG AAC CAT	GCA GCT GCT GTT GTA CCC AAA
Prox 1	GCG GTC CCG GGA TTC TT	GTT GCA CCA CAG AAT ATT CAA CAT C
VEGFR3	ATC GGC AAC CAT CTC AAC GT	GCT TTG GCG CCT TCT ACC A
eNOS	TGA TGG CGA AGC GTG TGA	GGG CCT GAC ATT TCC ATG AG
GAPDH	GGG AAG CCC ATC ACC ATC T	GCC TCA CCC CAT TTG ATG TT
β-actin	CGA GGC CCA GAG CAA GAG	CAC AGC CTG GAT GGC TAC GT
***Q-PCR Primer Sequences***		
Target Gene	Forward Primer	Reverse Primer
AC133	CCT CTG GTG GGC TGC TTC T	CAT CTC TCC GCC GCA TTT
NCAM	CAA CAT GGA GGG CAT TGT CA	CGA TCC GAG TAC GTC GTC TCA
CXCR4	GCC CTC GCC TTC TTC CA	CCC CGA GGA AGG CAT AGA G
Syndican 4	CCC AGG GCA GCA ACA TCTT	GCC GCC CAC GAT CAG A
Ephrin B2	GGA GGA CAC GGT AGG CTA TGG	CCT GGT GCG CAA CCT TCT
Eph B4	GAA GAG ACC CTG TTG AAC ACA AAA	CCT GAG GGT AAG TCA CCC ATT T
VEGFR3	ATC GGC AAC CAT CTC AAC GT	GCT TTG GCG CCT TCT ACC A

### Quantitative RT-PCR

Samples for quantitative RT-PCR were setup using the sybr green PCR master mix from Applied Biosystems (Foster City, CA) and primer pairs listed in table 1. Reactions were run on a ABI 7900HT PCR system. Results were analyzed using SDS 2.2 software and relative expression calculated using the comparative Ct method. cDNA from whole muscle was used to calibrate samples while GAPDH was used as an endogenous control. Error bars represent the standard deviation calculated from the triplicate analysis of each time point.

## Results

### Tissue staining reveals CD31^+^, Sca-1^+ ^and CD34^+^ vasculature

**Figure 1:**
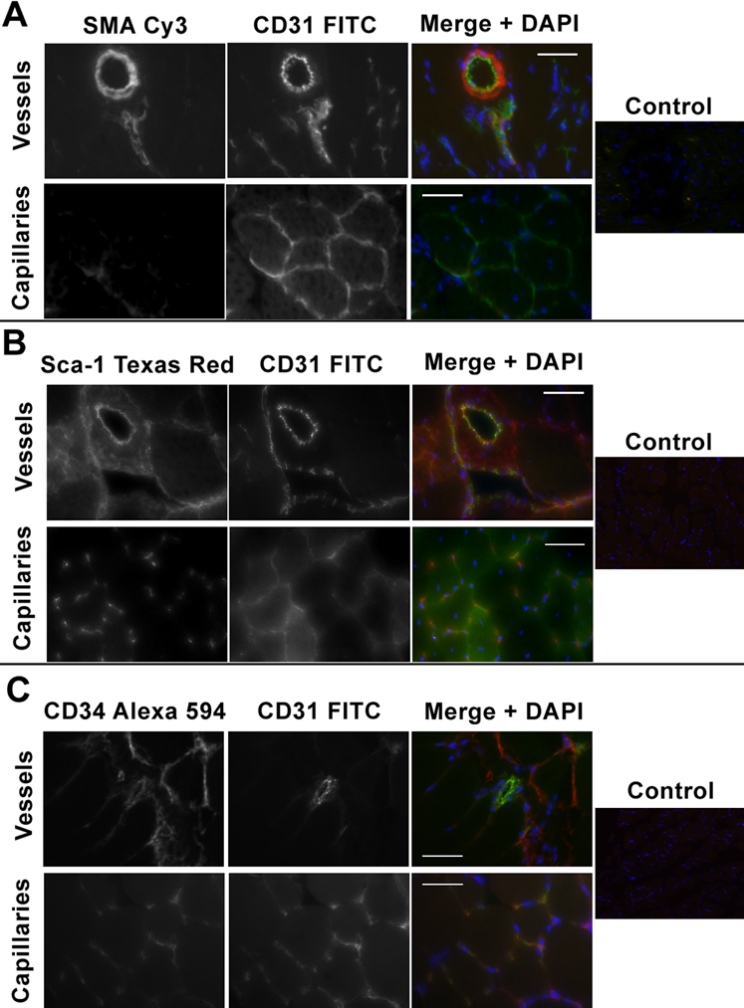
Endothelial cells of the skeletal muscle are CD31, Sca-1 and CD34 positive. Skeletal muscle tissue section staining for smooth muscle actin (SMA), CD31, Sca-1 and CD34 highlights vascular endothelial cells. All photographs were taken at 40x except controls which are at 10x. A, SMA Cy3 shows exclusive staining of the smooth muscles cells within veins and arteries and does not overlap with CD31 FITC staining which is localized in the endothelial lining of vessels. B & C, CD31 FITC positive vasculature is also positive for Sca-1 Texas Red and CD34 Alexa 594. Scale bar = 50 µm.

Muscle tissue staining for CD31 in parallel with SMA, Sca-1 and CD34 highlighted arterial, capillary and venular vessels (figure 1). The endothelial lining was clearly distinguishable from the smooth muscle layer of the vasculature by CD31 staining in the skeletal muscle (figure 1A). CD31 specifically stained endothelial cells in both arterial and venular vessels with no overlap of SMA staining, which exclusively depicted vascular smooth muscle cells (figure 1A). Capillaries while negative for SMA were also easily distinguishable by CD31 staining. Further staining indicated that muscle EC positive for CD31 were also positive for Sca-1 and CD34 (figures 1B & C). Though Sca-1^+^/CD31^−^ and CD34^+ ^/CD31^− ^cells were also present in the perivascular space, only cells positive for all three markers composed the vascular lining of arteries, veins and capillaries of the muscle. Sca-1^+^ and CD34^+^ cells negative for CD31 are perivascular cells negative for mature endothelial markers e.g. vWF (not shown).

### Sorted Sca-1^+^, CD31^+^, CD34^dim^ and CD45^− ^are skeletal muscle derived EC

**Figure 2:**
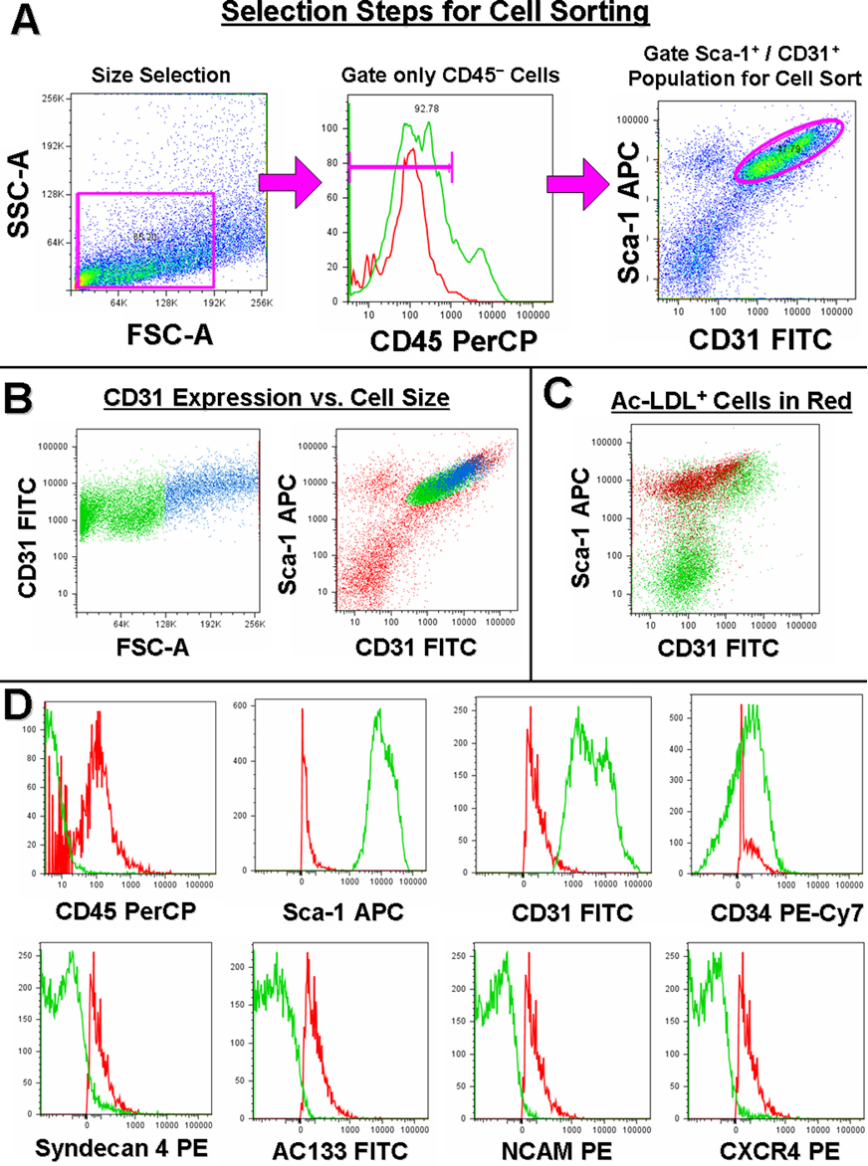
Flow-cytometry characterization of sorted EC. A, Stepwise selection for Sca-1^+^, CD31^+^, CD34^dim^ and CD45^− ^endothelial cells (EC) begins by first gating (pink box, left dot plot) on small and low granulated cells within a SSC-A and FSC-A dot plot. CD45^+^ cells are then identified and gated out by comparing the sample (green peak) with the unstained control (red peak) to reveal a Sca-1^+^ and CD31^+^ population of muscle EC within the CD45^− ^population (pink bar, middle histogram). The Sca-1^+^ (vertical)/CD31^+^ (horizontal) endothelial population (pink oval, right dot plot) which is dim for CD34 can then be gated for further analysis or cell sorting. B, Endothelial CD31 FITC expression correlates with cell size as FSC-A comparison indicates larger cells (blue) express more CD31. C, 45% of EC are positive for ac-LDL rhodamine uptake (red) within the entire CD45^−^ population (green) portrayed in Sca-1 APC (vertical) and CD31 FITC (horizontal). D, The endothelial cells defined as Sca-1^+^, CD31^+^ and CD34^dim ^are homogeneously negative for CD45 PerCP, positive for Sca-1 APC and CD31 FITC, and dim for CD34 PE Cy7 (green peaks) vs. unstained controls (red peaks). Analysis of separate aliquots from the same muscle preparations revealed EC lack Syndecan 4 PE, AC133 FITC, NCAM PE and CXCR4 PE (green peaks EC samples, red peaks unstained control).

Hematopoietic cells constitute a considerable fraction within the whole muscle mononuclear cell preparation. In addition, since some hematopoietic cells express Sca-1, CD31 or CD34, we first gated out CD45^+^ cells to remove any hematopoietic contamination (figure 2A). Muscle EC were then sorted as a distinct Sca-1^+^ and CD31^+ ^population that was dim for CD34. Sca-1^+^,CD31^+^ and CD34^dim^ cells comprised 30-55% of all CD45^− ^mononuclear cells isolated. 99.9% of cells sorted as Sca-1^+^,CD31^+^ and CD34^dim^ proved positive for vWF staining indicating this phenotype represents a pure population of EC. Side scatter analysis (figure 2B) of our sorted population revealed larger cells express higher levels of CD31, indicating its expression is relative to cell size. Interestingly, approximately 55% of sorted muscle EC were negative for ac-LDL uptake while muscle EC positive for ac-LDL comprised only 45% of all ac-LDL^+^/CD45^−^ cells (figure 2C). Further analysis confirmed sorted EC are collectively CD45^−^, Sca-1^+^, CD31^+^ and CD34^dim^ while being negative for Syndecan 4, AC133, NCAM and CXCR4 (figure 2D).

**Figure 3:**
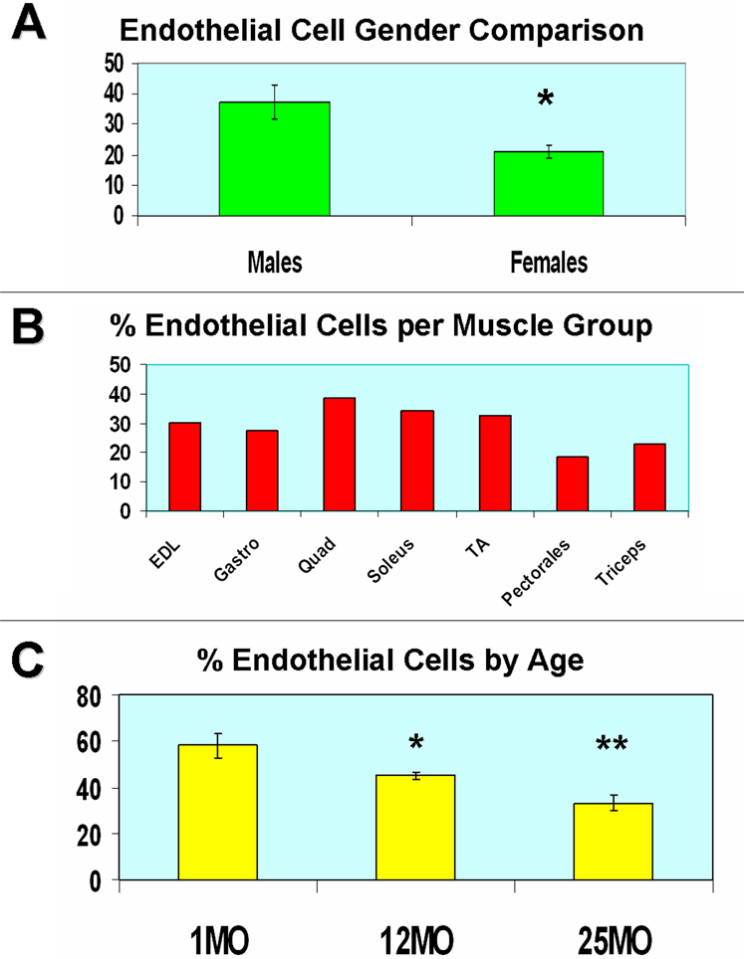
Muscle EC abundance between gender, muscle goups and age. A, Gender comparison of the percentage (y-axis) of EC within the CD45^−^ population between 2 MO mice (n = 3). * designates p<0.05. B, The percentage of EC within the CD45^−^ population among different skeletal muscles. C, Age comparison of the percentage of EC within the CD45^−^ population between 1, 12 and 24 MO mice (n = 3). * designates p<0.05 between 1 vs. 12 MO mice. ** designates p<0.05 between 12 vs. 25 MO mice. P values calculated by student t-test.

Gender comparisons performed at 2 months of age revealed that females have a lower percentage of muscle EC, 16% less EC as compared to males same age (figure 3A). Due to this gender variability we used only male mice for the duration of the study. Similar to the endothelial staining observed in different skeletal muscles the FACS phenotype of EC among different muscle groups was very consistent. However, there seems to be variation in the percentage of muscle EC not only between muscle groups but also with age (figure 3B & C). Although due to the limited number of individual muscle samples we did not statistically substantiate muscle group differences, hind limb muscles appear to have more EC vs. the upper body pectorals and triceps (figure 3B). Age comparisons revealed a statistically significant reduction with age as the percentage of muscle EC within the CD45^−^ population decreased an average of 26% from 1 to 25 MO mice (figure 3C).

### Sorted EC proliferated and expanded beyond 80 days in culture

**Figure 4:**
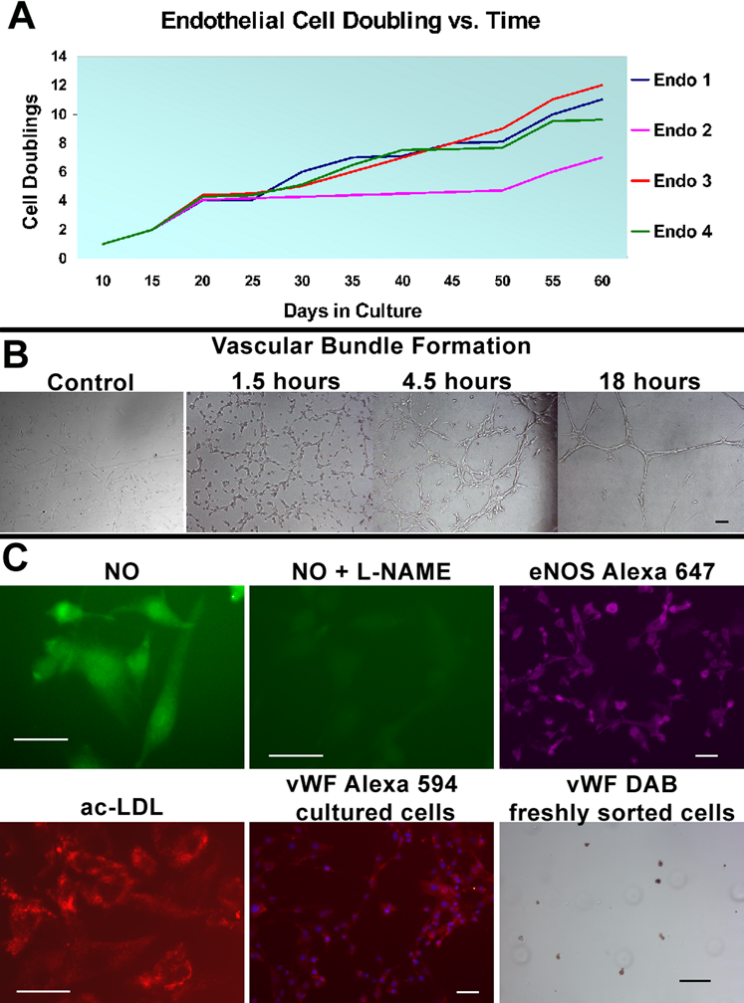
FACS sorted EC can be culture-expanded and preserve endothelial functions in culture. A, Growth rate comparison for muscle EC lines derived individually from four 25 MO C57BL6 males, cultured in DMEM with 10% FCS and 10 ng/ml VEGF for 60 days. B, Vascular bundle formation occurs rapidly within 18 hrs of cells being seeded on pure matrigel vs. controls without matrigel. Photographs were taken at 1.5 hour intervals and after 4.5 hours left overnight in culture. C, Top panel shows endothelial specific NO production. Left, muscle EC can produce NO as detected by green fluorescence emission of DAF-2T. Middle, NO production was inhibited by L-NAME. Right, muscle EC stained with rabbit anti-eNOS followed by anti-rabbit Alexa 647 show strong staining in endothelial caveolae. Bottom panel shows other functional assays as well as maintenance of vWF expression. Left, cultured muscle EC uptake ac-LDL- rhodamine (red fluorescence). Middle, skeletal muscle derived EC in culture retain vWF expression for more than 80 days as portrayed by anti-vWF with Alexa 594. Right, 99.9% of freshly FACS-sorted muscle EC stained positive for vWF immunoperoxidase with DAB (brown). Scale bar = 50 µm.

Initially we were unsuccessful culturing EC at atmospheric oxygen (20% O_2_). Therefore we attempted to grow them at physiological oxygen levels (5% O_2_). Freshly sorted muscle EC derived individually from four 25 MO C57BL6 males were cultured in DMEM-high glucose supplemented with 10% FCS and 10 ng/mL of VEGF. Medium was changed after the first 2 days and every four days afterwards. Under these conditions EC grew slowly during the first week achieving first passage after 10 days and afterwards steadily doubling every 4–6 days. EC looked very homogenous since early culture, grew in a monolayer and proliferated at a synchronous rate. Within 60 days in culture EC divided between 7–12 times (figure 4A). Muscle EC grown to subconfluency prior to passage were consistently viable beyond 80 days in culture.

### Cultured cells maintain endothelial phenotype and functional capabilities

Muscle EC tested at various times in culture maintained their functional capabilities and phenotype. Vascular tube formation occurred rapidly, completing within 18 hours of cells being plated on pure matrigel (figure 4B). Even at 1.5 hours, muscle EC appeared to be aligning and by 4.5 hours tube formation was observed. Cells were also capable of ac-LDL uptake and NO production while being positive for vWF staining even after 80 days in culture (figure 4C). Endothelial specificity of NO production was confirmed by L-NAME inhibition and positive staining for eNOS.

**Figure 5:**
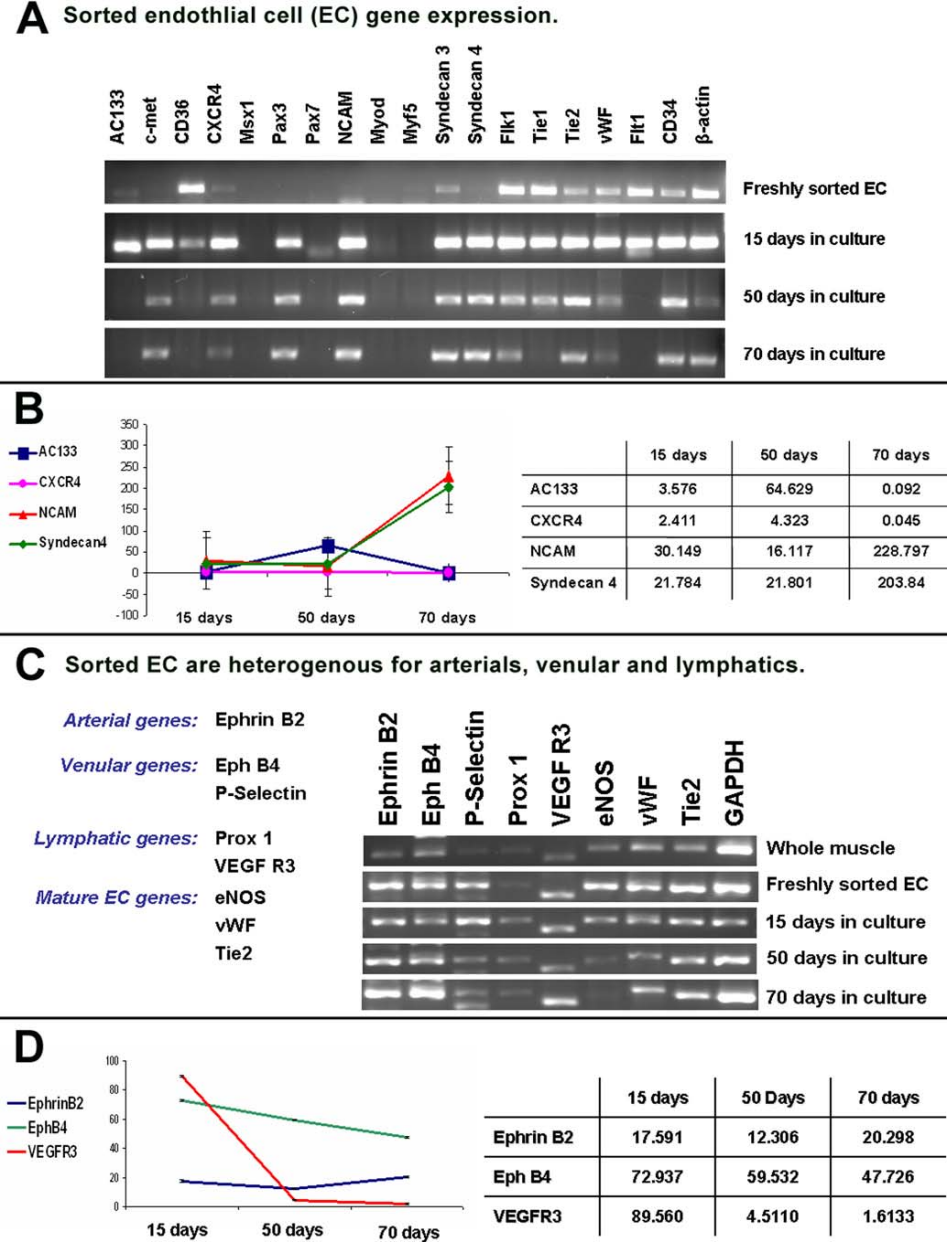
RT-PCR and Q-PCR of freshly FACS-sorted and cultured muscle EC reveals the expression of major endothelial markers, upregulation of certain adhesion markers in culture, and arterial, venular and lymphatic heterogeneity. A, RT-PCR results of freshly FACS-sorted and cultured EC indicates they maintain expression of common endothelial markers vWF, Flk-1 and Tie2 while lacking key myogenic markers Pax7, MSX 1, MyoD and Myf5. B, Q-RT-PCR of adhesion markers AC133, CXCR4, NCAM, and Syndecan 4 highlights significant increases of Syndecan 4 and NCAM expression after 50 days in culture whereas expression of AC133 decreases to baseline levels. C, RT-PCR analysis of arterial, venular, and lymphatic markers indicates sorted muscle EC comprise a hetergenous population of all three vascular origins that are maintained in culture. D, Q-RT-PCR of three specific arterial (Ephrin 2B), venular (EphB 4) and lymphatic (VEGFR3) markers confirms the maintenance of all three types of EC. Q-RT-PCR values are normalized to whole muscle from the same mouse sample. Error bars for this Q-RT-PCR are present but not clearly visible due to the standard deviations being <1.

RT-PCR of freshly sorted cells confirmed the expression of many endothelial markers such as vWF, Flk-1(VEGF-Receptor 2), Flt-1 (Fms-like Tyrosine Kinase-1), Tie 1 and Tie 2 (Tunica Internal Endothelial Cell Kinase 1 and 2), as well as the endothelial associated marker CD36 [21]. Although muscle EC express some markers associated with satellite cells such as Syndecan 3 and CD34 they were negative for definitive myogenic markers such as Pax7 (paired box gene 7), MSX1 (msh homeobox homolog 1), and muscle regulatory factors MyoD and Myf 5 (figure 5A) [22]–[25].

After 70 days in culture muscle EC maintained the expression of major endothelial markers while remaining negative for myogenic markers (figure 5A). Interestingly by RT-PCR muscle EC in culture appear to upregulate markers associated with adhesion, migration and mobilization such as c-met (Mesenchymal epithelial transition factor), CXCR4, Pax 3 (paired box gene 3), NCAM (neural cell adhesion molecule), Syndecan 3 and 4 [26]–[31]. Q-RT-PCR confirmed a significant upregulation of NCAM (48 fold increase) and Syndecan 4 (185 fold increase) beyond 50 days in culture while AC133 was transiently upregulated before 50 days in culture (figure 5B). In addition, late cultured muscle EC preserved functional activities such as vascular tube formation, ac-LDL-uptake and NO production.

### The FACS-sorted Sca-1^+^, CD31^+^, CD34^dim^ and CD45^− ^population contains arterial, venular and lymphatic endothelial cells that are maintained in culture

Although the Sca-1^+^, CD31^+^, CD34^dim^ and CD45^−^ phenotype represents a pure population of muscle EC, it is a heterogeneous collection of arterial, venular and lymphatic EC. To determine if selection occurs early in culture, we counted the number of cells that de-attached or died and found that only 12.4% of sorted EC are lost in culture within the first 24 hours of being sorted (data not shown). Endothelial progenitor cells (EPC) from blood are classically defined as the non-adherent cell population within the first 48 hours of culture in endothelial cell growth medium [32]. Thus non-adherent cells were removed 48 hours after initial plating to eliminate any EPC contamination.

To eliminate the possibility that selection of any endothelial subtype occurs in culture we compared the expression of various markers specific for all three EC subtypes and confirmed the presence and maintenance of arterial, venular and lymphatic EC throughout culture (figure 5C). Results indicated that freshly sorted cells contain and maintain expression of the arterial marker Ephrin B2, venular markers Eph B4 and P-Selectin, as well as lymphatic markers Prox1 and VEGFR3 [33]–[36]. Q-RT-PCR of Ephrin B2, Eph B4 and VEGFR3 confirmed the expression of arterial, venular and lymphatic specific genes throughout culture (figure 5D). Although, levels of Ephrin B2 mRNA (arterial specific marker) remained steady, EphB4 (venular specific) and VEGFR3 (lymphatic specific) decreased in long-term culture.

### Cultured muscle EC are angiogenic when transplanted in vivo

**Figure 6:**
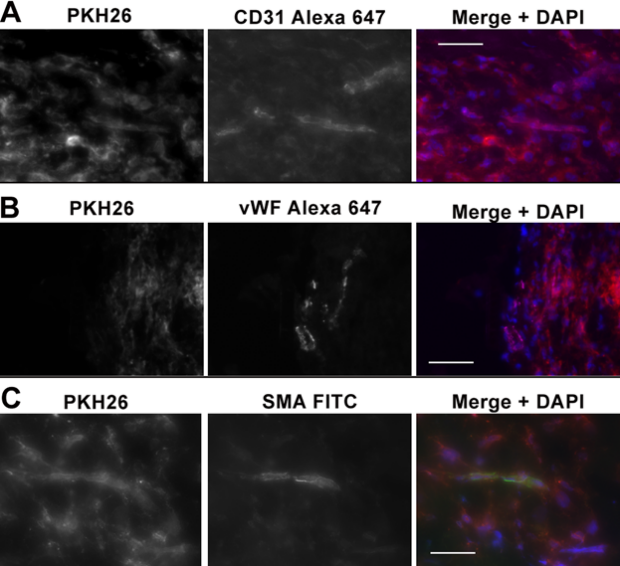
EC of the skeletal muscle form microvessels in matrigel injected subcutaneously over the dorsum. A–B, Tissue sections of the matrigel plug recovered from the dorsal skin of C57BL6 mice analyzed 14 days post injection reveal microvessel formation. Staining for anti-CD31 and anti-vWF followed with a secondary Alexa 647 antibody highlight mature vessels formed within the matrigel by injected cells (PKH26^+^). C, Conjugated FITC anti-SMA staining indicates the recruitment of endogenous smooth muscle cells in the formation of microvessels. Scale bar = 50 µm.

We first tested the angiogenic capacity of muscle EC with the widely used dorsal matrigel plug technique [20]. To track cells *in vivo*, muscle EC were labeled with PKH26, a red fluorescent dye that irreversible binds the cell membrane and has successfully been used for *in vivo* transplantation of EC [37]. 14 days after transplant PKH26^+^ EC formed new vessels that stained positive for CD31 and vWF. PKH26^+ ^EC made “bonafide” microvessels as endogenous smooth muscle cells, identified as SMA positive but PKH26 negative, were seen wrapping PKH26^+^ vessels (figure 6). Thus, we hypothesized that muscle EC may exhibit angiogenic potential if transplanted back into their tissue of origin, the skeletal muscle.

**Figure 7:**
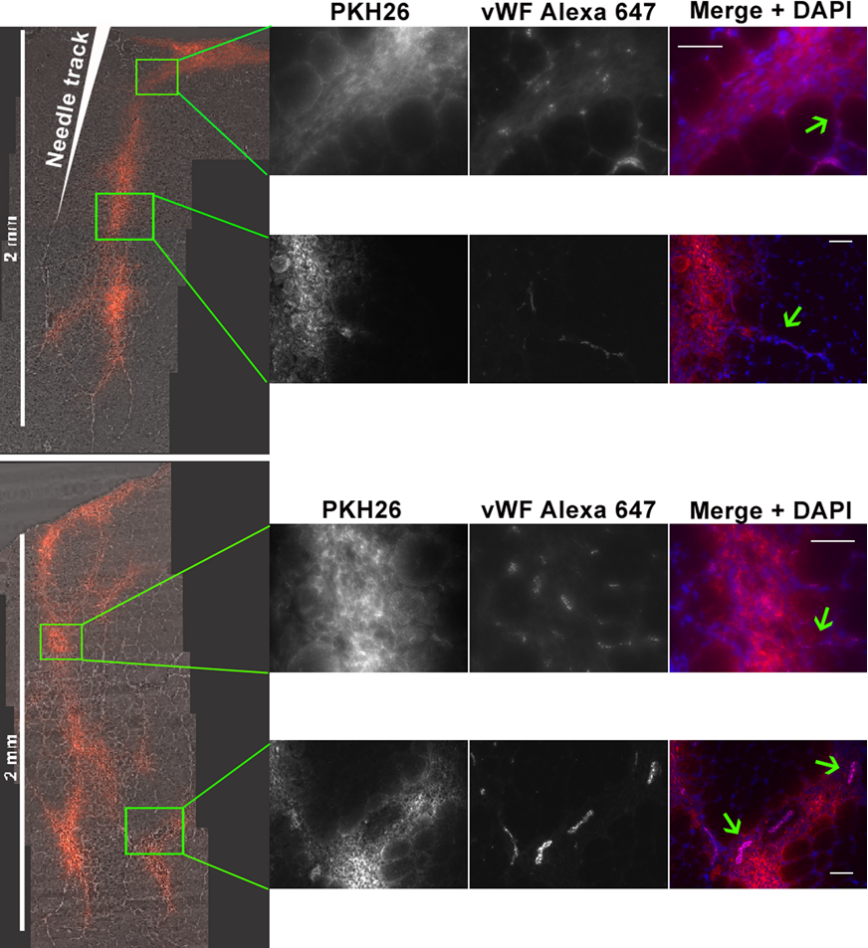
EC injected intramuscularly migrate, form new vessels and integrate with existing vasculature. Top, a montage of a region proximal to the injection site shows the needle track in the TA muscle. Bottom, a montage of a region approximately 80 µm distal to the injection site shows extensive migration beyond 1 mm from the needle track (white line). Montage represents an overlay of the PKH26 red fluorescence over bright field microscopy. Formation of new vessels and incorporation into existing vasculature is highlighted by anti-vWF staining in Alexa 647 (magenta). Green arrows point to areas where PKH26^+^ cells are making connections with or integrated into endogenous vessels. Scale bars = 50 µm.

14 days after direct injection into the TA, muscle EC seemed to have migrated beyond the needle track as new trails of PKH26^+^ cells were seen distal from the needle track forming new microvessels and incorporating into existing vasculature as indicate by detection of PKH26^+^ cells in vWF^+ ^vessels (figure 7). New vessels could be distinguished from pre-existing vessels by size, anatomic position and vessel wall thickness. The formation of new vasculature was primarily observed along paths of migration towards perivascular regions where transplanted EC made connections with and incorporate into existing vasculature. Interestingly, although PKH26^+^ EC were observed integrating and making connections with endogenous capillaries and venular vessels, we did not observe PKH26^+^ EC in major arteries. Due to lack of antibodies specific and reactive for lymphatic vessels in mouse skeletal muscle we were unable to determine if transplanted EC also contribute to lymphatic vessel formation or engraftment.

**Figure 8:**
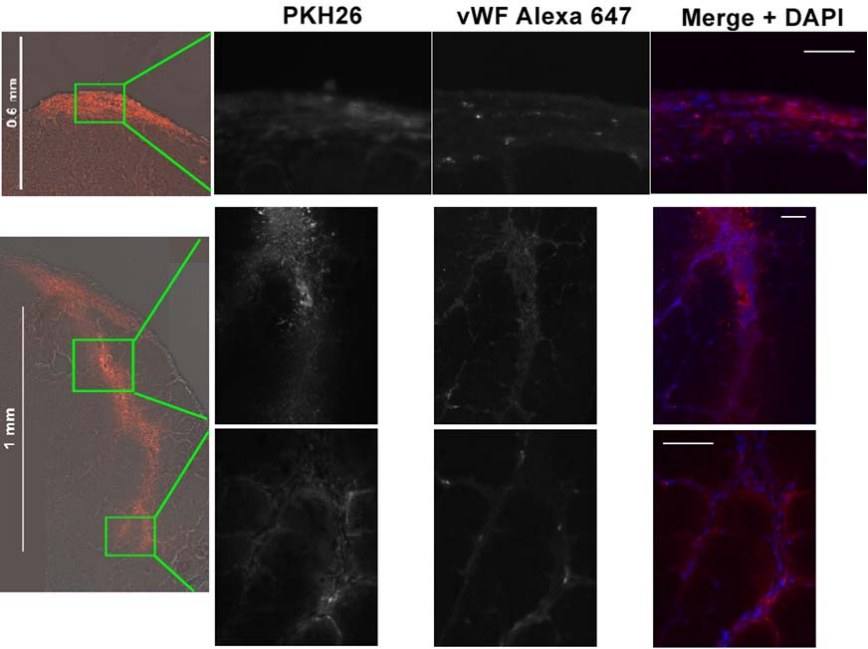
EC injected subcutaneously in matrigel migrate into the muscle. Top, a montage of the matrigel plug shows PKH26^+^ microvessels in the matrigel plug over the TA muscle. Bottom, A montage of an area approximately 80 µm distal shows migration of PKH26^+^ EC. EC invaded the TA from the matrigel plug and formed new microvessels along their migration path and also integrated into existing vessel. Staining with anti-vWF in Alexa 647 (magenta) highlights EC angiogenic capability within the matrigel plug and muscle. Scale bars = 50 µm.

To better study the *in vivo* migration capacity of cultured muscle EC, PKH26 stained EC embedded in matrigel were injected subcutaneously over the TA. As expected many PKH26^+ ^EC formed new microvessels within the matrigel plug, similar to what was seen in the dorsal matrigel plugs. Moreover, significant migration into the muscle was observed in sections distal from the matrigel plug (figure 8). In this transplant study, it was obvious that PKH26^+ ^EC migrated at least 1 mm deep into the muscle tissue from matrigel plug. In summary, these results consistently proved that cultured muscle EC can form and integrate in microvessels *in vivo* as vWF^+^/PKH26^+^ vasculature was observed in all three sets of transplants.

### Freshly FACS sorted Sca-1^+^, CD31^+^, CD34^dim^ and CD45^− ^cells can from microvessels upon direct transplantation into the skeletal muscle

**Figure 9:**
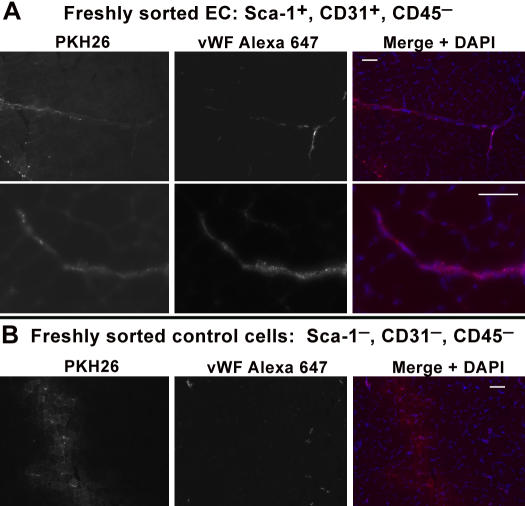
Freshly sorted EC injected intramuscularly also form new vessels and integrate with existing vasculature vs. control cells. A, Tissues sections from mice injected intramuscularly and examined 14 days post injection reveals freshly sorted PKH stained EC also have angiogenic capability. Although freshly sorted EC did not migrate as far as cultured EC several PKH^+^/vWF^+^ vessels highlighted by anti-vWF staining with Alexa 647 (magenta) were visible beyond the point of injection. B, Contrary to the capability of freshly sorted and cultured EC to form new vessels and integrate with existing ones, control cells sorted as Sca-1^−^, CD31^−^ and CD45^−^ injected under the same conditions did not exhibit any angiogenic potential. Scale bars = 50 µm.

To test whether such a robust angiogenic capability was an artifact of culture conditions, freshly sorted EC (Sca-1^+^, CD31^+^ and CD45^−^) vs. freshly sorted control cells (Sca-1^−^, CD31^−^ and CD45^−^) were directly injected into separate TAs of uninjured C57BL6 mice and examined 14 days post transplant. Although we observed that freshly sorted EC did not migrate so extensively as cultured EC, vWF staining revealed they did form new microvessels and incorporated into existing vasculature vs. control cells which were not at all angiogenic (figure 9). Therefore freshly sorted muscle EC defined as Sca-1^+^, CD31^+^ and CD45^−^ cells are angiogenic, mature EC. To our knowledge this is the first report of successful transplantation of freshly FACS-sorted EC with angiogenic capacity.

## Discussion

Here we describe a new method utilizing multicolor flow cytometry to isolate pure populations of EC from the skeletal muscle based on the antigens Sca-1, CD34, CD31 and CD45. Immunofluoroscence staining revealed Sca-1, CD31 and CD34 expression in the vasculature of skeletal muscle. Sca-1 is predominantly a marker of hematopoietic stem cells, although it can be expressed by other cells including EC [38]–[40]. CD34 is also a marker of hematopoietic stem cells and EC [41], [42]. CD31 is expressed by EC in multiple types of tissue and has even been previously described for EC isolation [17], [19], [30], [43]. Though CD31 has been widely characterized in EC from various tissues, to our knowledge this is the first study to show CD31 expression in EC derived from skeletal muscle. Because many of these markers are also expressed by hematopoietic cells, we used CD45 to eliminate hematopoietic cells that may also express Sca-1, CD31 or CD34.

FACS analysis revealed varying levels of CD31 expression based on size, bigger EC express higher CD31 levels. Cell size may differ between different types of microvascular EC. Also, heterogeneity within the muscle EC population was observed by ac-LDL uptake which stains only 45% of the Sca-1^+^, CD31^+^, CD34^dim^ and CD45^−^ cells. In addition, muscle tissue staining for Sca-1, CD31, and CD34 confirmed these markers are expressed in arterial, including capillaries, and venular EC. In retrospect our flow cytometry approach does not distinguish among different EC subtypes, however has proven very specific for only EC as the majority of sorted cells are positive for vWF (>99.9) and other endothelial markers. Therefore the Sca-1^+^, CD31^+^, CD34^dim^ and CD45^−^ population although free of non-endothelial cells contains many endothelial subsets that differ in size, ac-LDL uptake and other endothelial functions, perhaps indicating ontological or functional differences among arterial, capillary and venular EC.

RT-PCR of arterial, venular and lymphatic genes indicates that all three EC subtypes are present in the freshly sorted population. Unfortunately we were unable to find antibodies applicable for flow cytometry that prove specific for arterial, venular and lymphatic EC. With the coming of new endothelial specific markers it may be possible to distinguish arterial, venular and lymphatic EC. Nonetheless changes in global muscle microvascular endothelium can be studied using this current method. For instance, the differences observed between age and gender groups are very interesting and intriguing. However, further studies are needed to understand the significance of such variations.

There are many diseases associated with endothelial dysfunction. The causes and mechanisms of endothelial dysfunction are not well understood. Oxidative stress, alterations in the insulin signaling, and NO signaling have been associated with endothelial dysfunction [44]–[48]. However, many of these studies are limited by changes or artifacts acquired in culture. Our flow cytometry approach offers the advantage to study freshly dissociated muscle EC prior to any *in vitro* manipulation and thus we believe that this approach is suitable and appropriate to study the molecular mechanisms involved in endothelial dysfunction. Traditionally, primary mouse endothelial cells are very difficult to grow *in vitro* and under ordinary culture conditions (20%O_2_) muscle EC failed to grow. In turn, by using culture condition closer to physiological conditions (5%O_2_) we can maintain and expand this muscle EC population for *in vitro* or *in vivo* studies.

Functional assays, staining and RT-PCR indicate muscle EC sorted as Sca-1^+^, CD31^+^, CD34^dim^ and CD45^−^ retain their functional capabilities and phenotype even after multiple passages in culture. Though c-met, CXCR4, Pax 3, NCAM, Syndecan 3 and 4 were upregulated in culture, it has been previously reported that *in vitro* EC upregulate such genes in response to VEGF [49], [50]. Moreover, the expression of endothelial related genes vWF, Tie2 and Flk-1 remained consistently positive throughout culture.

The expression of arterial, venular and lymphatic markers was detected in freshly FACS-sorted samples and maintained in culture up to 70 days, indicating that all three types of endothelial cells (arterial, venular and lymphatic) are included in our sorted starting population and their phenotype is maintained in culture. Although expression of VEGFR3 (a lymphatic marker) is drastically decreased in late culture, we were unable to determine if lymphatic EC are lost in late culture due to lack of specific and reactive antibodies for lymphatic vessels of the mouse skeletal muscle. Nonetheless, mature endothelial markers and endothelial functions examined in our study remained consistent from isolation to culture beyond 80 days. Therefore sorted muscle EC can be maintained and expanded for transplant or long term *in vitro* studies.

Cultured muscle EC were able to form microvessels when transplanted subcutaneously in matrigel plugs. In addition, when cultured EC were directly injected intramuscularly in syngeneic mice, microvessel formation was observed as well as extensive migration and integration into existing vessels along and beyond the injection track. To better study the capacity of these cultured EC to migrate within the muscle, we transplanted cultured EC embedded in matrigel subcutaneously over the TA without penetrating the muscle. As expected we observed formation of microvessels in the matrigel plug but also migration of EC into the muscle parenchyma. These cells not only migrated and made microvessels in the perimysium and endomysium but also integrated into existing vessels within the skeletal muscle. In addition, we did not observe any signs of rejection or inflammation in the transplanted muscle. Thus we report here a new route for successful transplantation of muscle EC by direct intramuscular injection or subcutaneous delivery with matrigel.

When freshly FACS-sorted cells are directly transplanted into skeletal muscle engraftment into existing vessel and formation of newmicro vessels was also observed. To our knowledge this is the first report of direct transplantation of pure freshly sorted endothelial cells with angiogenic capacity. We believe this approach will prove to be very beneficial and advantageous for clinical application as high number of endothelial cells from fresh muscle (30–55% of all mononuclear muscle cells) can be obtained for direct transplantation circumventing the need of *ex vivo* expansion which is a major limitation of stem cell transplants for therapeutic angiogenesis.

Because these cells showed tremendous angiogenic potential and migration upon direct transplantation in uninjured skeletal muscle we are currently studying the potential mechanism(s) of chemoattraction. Although Sca-1 is not expressed in human cells, all the other markers used for the isolation of mouse EC cells are widely expressed by human cells and thus we believe this protocol could be applied to isolate EC from human skeletal muscles. Furthermore by making it possible to isolate pure EC from the skeletal muscle, this method can be used to advance the study of EC biology and role in normal and diseased muscle.

The comparison of muscle EC with other organ endothelial cells (Table 2) reveals that muscle EC share many expression markers with other tissue microvascular cells (e.g. dermal EC, sinusoidal liver cells, pulmonary and kidney capillary EC) such as CD36, Tie1, Tie2, vWF, Flt-1, CD34, Sca-1and CD31. However muscle EC express some markers not previously reported by other endothelial cells. Syndecan 3 is expressed in freshly sorted and cultured muscle EC and Syndecan 4, NCAM and Pax3 are expressed in cultured muscle EC. Thus these markers may delineate the origin of muscle EC and may be useful to identify and distinguish muscle derived endothelial cells from other organ endothelial cells. However, a more extensive study of the expression of these markers in other organ endothelial cells is needed to determine that these markers are exclusive and ontogenic of muscle endothelial cells.

**Table 2:** Muscle vs. other organ derived endothelial cell (EC) gene expression.

	AC133	c-met	CD36	CXCR4	Pax 3	Pax 7	NCAM	Syndecan 3	Syndecan 4	Flk1	Tie1	Tie2	vWF	Flt-1	CD34	Sca-1	CD31	CD45
Sorted Muscle EC	−	−	+	−	−	−	−	+	−	+	+	+	+	+	+	+	+	−
Cultured Muscle EC	+	+	+	+	+	−	+	+	+	+	+	+	+	+/−	+	+	+	−
EPC- Muscle												+[57]				+[57]	−[57]	−[57]
EPC-Blood	+[60]									+[61]	+[62]	+[61]	+[63]		+[60]	+[62]	+[61]	+/−[58]
Dermal EC			+[21]								+[64]	+[64]	+[64]	+[64]	+[64]		+[64]	−[64]
Liver Sinusoid EC			+[21]							+[65]		+[66]	+[67]	+[68]	+[67]	+[40]	+[67]	
Pulmonary Macrovascular EC			−[21]							+[65]	+[69]	+[66]		+[68]		+[39]		
Pulmonary Capillary EC			+[21]							+[70]	+[71]	+[66]	+/−[67]		+[67]	+[39]	+[67]	
Spleen Sinusoid EC			+[21]										+[67]		−[67]		+[67]	
Kidney Glomerular and Capillary EC			+[21]							+[65]	+[69]	+[66]	+/−[67]	+[68]	+[67]		+[67]	

+ or − indicates expression of listed genes (x-axis) relative to each EC type (y-axis).

+/− indicates expression diminishes in culture or has been reported to be and not to be expressed.

Sorted and Cultured EC expression is based on RT-PCR results portrayed in figure 5.

Sources for reported gene expression by other organ derived EC are included within the brackets.

Blank space = expression has not been reported.

Recently several stem cell populations from the skeletal muscles with endothelial and myogenic potential have been characterized [51]–[56]. Some of these populations, e.g. mesoangioblasts, express immature endothelial markers and myogenic markers [51]. These cells have been localized in the perivascular tissue. In contrast, the endothelial population from the skeletal muscle that we describe here expresses mature endothelial markers while lacking expression of myogenic markers. While muscle EC exhibit tremendous angiogenic capacity upon transplantation no myogenic potential was observed *in vivo*. In addition, staining for Sca-1, CD34, CD31 and SMA highlights their localization in the vessels (both arterial, including capillaries, and venular) of the skeletal muscle. Thus these muscle EC are clearly distinguishable from the perivascular a.k.a. pericytes with putative myogenic potential [51], [53], [54].

Moreover, a recent report describes the isolation of EPC from skeletal muscle as well as other tissues [57]. These endothelial precursors express immature endothelial markers while lacking expression of mature endothelial markers including CD31 (table 2). Although EPC from cord and peripheral blood express CD31 and other mature EC markers, they also express hematopoietic markers such as CD45 and AC133 (table 2) [58], [59].

Although we cannot rule out that our muscle EC FACS-sorted population may contains rare endothelial precursors, the EC population in our multicolor flow cytometry assay is defined as CD31 positive cells and the vast majority of the freshly sorted EC express vWF and eNOS. Furthermore, our culture method seems to select out EPC as we remove non-adherent cells after 48 hours of initial culture and mature EC maintain a consistent and homogenous morphology, proliferation rate and phenotype through long-term culture [32]. Thus, muscle EC described in our method constitute a mature endothelial population derived directly from the microvasculature of the muscle that can be maintained in culture. We believe that our approach of first identifying markers expressed by different muscle populations based on their anatomic position and applying this knowledge to separate the cell populations by flow cytometry will be useful to understand the relationship of “bonafide” endothelial cells with endothelial precursor cells or multipotent cells of the skeletal muscle.

In summary we report a highly reproducible method for sorting skeletal muscle EC utilizing the cell surface antigens Sca-1, CD31, CD34 and CD45. We have successfully characterized the Sca-1^+^, CD31^+^, CD34^dim^ and CD45*^−^* population as functional EC from multiple mice ages ranging from 1–25 months old. To our knowledge this is the first report of isolation and characterization of EC from the skeletal muscle. Furthermore this is the first report that describes direct transplantation and subsequent vascularization of skeletal muscle by mature EC derived from the same tissue.
